# Clozapine prescribing in COVID-19 positive medical inpatients: a case series

**DOI:** 10.1177/2045125320959560

**Published:** 2020-09-13

**Authors:** Matthew Butler, Felicity Bano, Marilia Calcia, Isabel McMullen, Chun Chiang Sin Fai Lam, Laura Jane Smith, David Taylor, Siobhan Gee

**Affiliations:** Institute of Psychiatry, Psychology, and Neuroscience, King’s College, London, SE5 8AF, UK and South London and Maudsley NHS Foundation Trust, London, UK; South London and Maudsley NHS Foundation Trust, London, UK; Institute of Psychiatry, Psychology and Neuroscience, King’s College, London, UK; South London and Maudsley NHS Foundation Trust, London, UK; South London and Maudsley NHS Foundation Trust, London, UK; South London and Maudsley NHS Foundation Trust, London, UK; King’s College Hospital, London, UK; South London and Maudsley NHS Foundation Trust, London, UK; Institute of Pharmaceutical Sciences, King’s College London, London, UK; South London and Maudsley NHS Foundation Trust, London, UK

**Keywords:** COVID-19, SARS-CoV-2, clozapine, schizophrenia, psychosis

## Abstract

There is both uncertainty regarding the safety of clozapine in COVID-19 patients owing to limited published data and a lack of consensus on continuing clozapine in patients with severe respiratory infections. COVID-19 is known to induce an acute immune response which can affect haematological parameters associated with clozapine monitoring, and systemic infection may reduce clozapine clearance. Clozapine, which has been associated with worse outcomes in some pneumonias, may in theory worsen outcomes in COVID-19. Despite these concerns, there are some data to indicate it is safe to continue clozapine in COVID-19 infection. In this retrospective case series, we describe our experiences of clozapine prescribing and disease progression of eight SARS-CoV-2 positive patients on medical wards in a major London teaching hospital. In four cases clozapine was stopped during the hospital admission. A COVID-19 pneumonia developed in four patients: three of these required intensive care unit admission for an average of 34 days. At the time of writing, three patients had died (two directly from COVID-19 pneumonia), two remained in general hospital wards, two were recovering in the community and one had been transferred to an inpatient psychiatric hospital. Follow-up length varied but in each case was not more than 104 days. Delirium was the most common adverse neuropsychiatric event, and in one case a relapse of psychosis occurred after cessation of clozapine. This retrospective case series illustrates the safe use of clozapine during COVID-19 infection. Our experiences suggest that consideration should be made to continuing clozapine even in those most unwell with COVID-19. We also identify areas which require larger scale hypothesis-testing research.

## Introduction

SARS-CoV-2 infection, which first emerged in China at the end of 2019, has become a worldwide pandemic. Most individuals with COVID-19 (the syndromic illness cause by the virus) have a mild illness and respiratory symptoms predominate. Despite this, there is significant multi-organ involvement in those with severe disease.^1^

There are currently limited data on the characteristics and outcomes of patients taking clozapine with severe COVID-19 admitted to general hospitals. There have been case reports of successfully continuing clozapine treatment in mildly unwell COVID-19 patients in acute inpatient psychiatric wards.^2^ However, some authors have suggested that the inflammation associated with COVID-19 infection has the potential to precipitate clozapine toxicity,^3,4^ and that as clozapine has been associated with increased risk of pneumonia in general, it may also increase the risk of pneumonia in patients infected with COVID-19.^5^ As well as this, data have indicated that the inflammatory cytokines released during infections can inhibit CYP1A2 enzymes, potentially causing clozapine toxicity, with the risk possibly increasing in relation to the severity of inflammation.^6,7^ This may also be true for COVID-19.

Although it is likely to be challenging to commence clozapine therapy during the COVID-19 pandemic, current advice is to continue clozapine in acute COVID-19 illness,^8^ and in community patients without COVID-19 infection already prescribed the medication.^9,10^ There are provisional guidelines on the rationale for continuing or discontinuing clozapine^8^ and data are emerging on successful continuation of clozapine in the community during the COVID-19 pandemic.^11^

The lack of experience and published data may lead to uncertainty in decisions on the use of clozapine in severe COVID-19 cases. Here we describe eight consecutive patients admitted to medical wards in a large acute London hospital who were prescribed clozapine and developed COVID-19 infection.

## Methods

We collected data from patients using a retrospective observational case series design. Information was obtained from electronic clinical notes. Patients were included if they were admitted to inpatient acute medical wards (i.e. not a psychiatric hospital) in a large university teaching hospital in London in the period 16 March–1 May 2020, and had laboratory-confirmed positive SARS-CoV-2 infection using a reverse-transcriptase polymerase chain reaction assay for SARS-CoV-2 ribonucleic acid on nasopharyngeal swab, and were prescribed clozapine prior to or during admission. Included patients did not necessarily initially present with COVID-19 symptoms, nor were they all COVID-19 positive upon admission.

Absolute neutrophil count, troponin and C-reactive protein (CRP) were obtained from blood sample results performed at the laboratory associated with the hospital. Renal function is displayed as estimated glomerular filtration rate (eGFR), which was obtained through laboratory calculations based on the patient’s creatinine. Length of stay was included only if the patient had been discharged at the time of writing. Follow-up time was taken from either admission, COVID-19 positive swab date or referral to psychiatric liaison (depending on which was most relevant for each patient) until time of writing. The details of each case are described below.

Written consent for publication was sought from patients or a suitable relative if deceased. Order of preference for consent was face-to-face with written consent, telephone consent with subsequent posted written consent and, finally, telephone consent alone. Obtaining written consent was not always possible in part due to the restrictions imposed during the pandemic. Where consent was refused or the patient was felt to lack capacity, the case was sufficiently anonymised in line with journal policy, and narrative summaries were not included in these cases. Ethical approval for this series was obtained directly from the hospital as a service evaluation.

## Results

Eight patients were identified (five women), with a mean age of 62 years (Table 1). Most patients had been maintained on clozapine for many years prior to their presentation. Average clozapine dose on admission was 253.6 mg per day. Four patients developed a COVID-19 pneumonia: three of these required intensive care unit (ITU) admission, for an average of 34 days. At the time of writing, three patients had died (two directly from COVID-19 pneumonia), two remained in general hospital wards, two were recovering in the community and one had been transferred to an inpatient psychiatric hospital.

**Table 1.** Summary of clozapine management, clinical outcomes and clinical variables.

ID	Total daily clozapine dose on admission	Clozapine stopped	Complications	Outcome	Comorbidities	Ethnicity	Smoking status	Length of stay (days)	Follow-up duration (days)
**A**	600 mg	Yes – held <24 h then restarted at 400 mg ON	Delirium	Died	T2DM, COPD, HTN, anaemia, BEN, essential thrombocytopenia, hypercholesterolaemia, GORD, gout	Black	Current (vape)	Four	Four
**B**	350 mg	Yes – held <24 h then restarted at usual dose	Nil noted	Discharged to mental health unit	T2DM, anorexia	White	Non-smoker	Three	Three
**C**	75 mg	Yes – held on admission. Retitrated during admission to final dose of 200 mg BD	Nil noted	Discharged home	Thalassemia, cataracts, T2DM, hypercholesterolaemia	White	Current	Seven	Seven
**D**	300 mg	No, however required IM clozapine administration to maintain continuous therapy	Delirium, ITU, intubation	Died	Ischaemic bowel, renal failure, septic cardiomyopathy, endocarditis, asthma, SVC thrombus during admission; these mostly pre-dated SARS-CoV-2 infection	Black	Current	69	Three
**E**	250 mg	No – reduced to 80% of baseline dose	Delirium, ITU, intubation	Ongoing hospital care	T2DM, psoriatic arthropathy, overactive bladder	White	Current	–	49
**F**	300 mg	No – reduced to 66% of baseline dose	Delirium	Died	T2DM, COPD, HTN, hypothyroidism, osteoporosis	White	Current	Six	Six
**G**	450 mg	Yes – stopped on admission, retitrated *via* NG 2 months later	Delirium, ITU, intubation	Ongoing hospital care	T1DM, COPD, pancreatomy and splenectomy, depression	White	Current	−	50
**H**	250 mg	Yes – stopped then reiterated *via* NG 19 days later	Delirium, ITU, intubation	Discharged to rehabilitation ward	HTN, T2DM	Black	Non-smoker	93	104

BEN, benign ethnic neutropenia; COPD, chronic obstructive pulmonary disease; GORD, gastro-oesophageal reflux disease; HTN, hypertension; IM, intramuscular; ITU, intensive care unit; NG, nasogastric; SVC, superior vena cava; T1DM/T2DM, type I/II diabetes mellitus.

### Patient A

Patient A was a male 62-year-old with schizoaffective disorder who had been detained under Section 3 of the Mental Health Act 1983 in a psychiatric rehabilitation ward for four years prior to admission. He was managed on clozapine 200 mg OM and 400 mg ON, which he had taken since late 2018. He was additionally prescribed amisulpride 400 mg BD. Physical medications included amlodipine 10 mg OM, clopidogrel 75 mg OM, colecalciferol 800 units OM, sodium docusate 200 mg BD, ferrous sulphate 200 mg BD, gliclazide 160 mg BD, omeprazole 40 mg OM, ramipril 10 mg OM and tamsulosin MR 400 mg OM.

He was admitted to acute inpatient medical care after being noted to have increased confusion and thought disorder, pyrexia (39.7°C), hypoxia (oxygen saturations 86% on room air) and rigors. His chest X-ray and bloods (neutrophils 1.64 × 10^9^/L, lymphocytes 0.75 × 10^9^/L, CRP 45 mg/L) were suggestive of COVID-19 pneumonia. He was swabbed for SARS-CoV-2 and treated empirically with intravenous antibiotics to cover for a bacterial infection.

On admission, his morning dose of clozapine was held, but his evening dose continued at 400 mg ON. On day 2 a positive SARS-CoV-2 swab result was returned. He had been consistently pyrexial and short of breath during this time and was commenced on oxygen supplementation. His oxygen requirements increased over the following 24–48 h with deteriorating saturations. In the early hours of day 4 he clinically deteriorated and arterial blood gas showed type I respiratory failure (pO2 6.8 kPa). It was concluded that he would not benefit from invasive ventilation due to his significant medical comorbidities and likely poor prognosis. He died of COVID-19 pneumonia after a few hours of symptomatic treatment.

### Patient D

Patient D was 59-year-old woman with refractory schizophrenia who was maintained in the community on clozapine 100 mg OM 200 mg ON. She stopped taking this and was retitrated on admission. She was admitted to the general hospital for several months due to hospital-acquired pneumonia, ileus secondary to possible ischaemic bowel, and thromboembolic disease including a superior vena cava thrombosis, and renal failure treated with haemodialysis. Each of these medical comorbidities pre-dated her COVID-19 swab. Her clozapine dose had been switched to intramuscular administration due to swallowing difficulties, and her dose was accordingly reduced to 150 mg OD.

She was swabbed for SARS-CoV-2 late in her long admission after spiking temperatures of 39.0°C with increased oxygen requirements with raised inflammatory markers. At this point she was felt to be near the end of life due to her significant medical comorbidities, with COVID-19 symptoms overlaying her already poor medical status. She remained hypotensive, tachycardic and febrile and a venous blood gas indicated metabolic acidosis. She died 3 days later; her cause of death was myocardial infarction with COVID-19 as a contributing factor.

### Patient E

Patient E was a 62-year-old man with a diagnosis of bipolar affective disorder who tested positive for SARS-CoV-2 after developing a dry cough. He self-isolated as per governmental guidance. He continued his clozapine dose of 250 mg ON, citalopram 10 mg and sodium valproate 900 mg OM + 500 mg ON. Physical medications included aspirin 75 mg OD, atorvastatin 20 mg ON, bisoprolol 2.5 mg OD, metformin 1g BD, ranitidine 150 mg OD, folic acid 5 mg OD and methotrexate 12.5 mg weekly. On day 5 he was admitted to hospital after developing confusion, weakness and shortness of breath.

He was lymphopoenic (0.69 × 10^9^/L) and haemodynamically unstable on admission with signs of sepsis, as well as severe hypernatraemia (163 mmol/L). He was diagnosed with severe COVID-19 pneumonia and required intubation for mechanical ventilation on day 8 post developing symptoms. He was continued on a reduced dose (200 mg ON) regimen of clozapine given his smoking status in the community.

He was extubated on day 14, and a steady state trough clozapine serum concentration taken at this point was 550 ng/mL. He developed a significant ITU delirium, and haloperidol (2.5–5 mg) and lorazepam (0.5–2 mg) were prescribed as required (PRN), as was an IV infusion of clonidine. His PRN antipsychotic was then briefly switched to quetiapine; however, this was stopped after a few days. Sodium valproate was continued.

He was stepped down from ITU on day 25, and clozapine serum concentration on this day was 430 ng/mL; clozapine dose remained unchanged. At the time of writing, his delirium had fully resolved and he was progressing well.

### Patient F

Patient F was a 75-year-old woman with refractory schizophrenia, maintained on 300 mg OD clozapine. She was a lifelong heavy smoker with known chronic obstructive pulmonary disease (COPD). Regular physical health medications included levothyroxine 50 µg, carbocysteine 375 mg TDS, metformin 500 mg OD and Relvar Ellipta 184/22 µg OD.

She was admitted after being found on the floor of her accommodation with coughing and shortness of breath. Bloods on admission showed a lymphopenia (0.74 × 10^9^/L). Due to her smoking status, her clozapine dose was reduced to 200 mg OD upon admission. She tested positive for SARS-CoV-2 and was diagnosed with infective exacerbation of COPD and COVID-19 pneumonia with acute respiratory distress syndrome. She required non-invasive oxygen supplementation, with target saturations of 88–92%, and her oxygen requirements initially improved.

On day 4 she clinically deteriorated, with increased oxygen requirements, and became drowsy with type I respiratory failure. She was given symptomatic end-of-life treatment and died six days after admission.

### Patient G

Patient G was a 57-year-old woman with a diagnosis of treatment resistant schizophrenia who was admitted from a residential unit. She was prescribed clozapine 150 mg OM and 200 mg ON, aripiprazole 10 mg OM, lithium carbonate MR 400 mg ON, and sertraline 200 mg OM. She had stopped taking her medication for a few days prior to admission.

She was admitted to acute inpatient medical care with hypoxia, haemodynamic instability, and bloods suggestive of COVID-19 (oxygen saturation 94% on 10 L oxygen supplementation, CRP 122 mg/L, white blood cells 14.2 × 10^9^/L, neutrophils 10.3 × 10^9^/L). On day 1 she had a positive nasopharyngeal swab for SARS-CoV-2 and was diagnosed with type I respiratory failure (pO2 14.2 on 15 L of supplemental oxygen) secondary to COVID-19 pneumonia. On day 2 she was transferred to ITU due to increasing oxygen requirements and was intubated for mechanical ventilation. Psychotropic medications were not restarted.

On day 19, steps were taken to retitrate her psychotropic medications with an aim of facilitating future ventilator wean, and clozapine was given *via* her nasogastric (NG) tube. Clozapine titration was started at 12.5 mg/day, increasing in steps of 12.5 mg daily for the first 4 days, then increasing by 25 mg daily for the following 2 weeks until a dose of 450 mg was reached. Her lithium continued to be held due to poor renal function, (eGFR 12 mL/min). During this time, she had significant episodes of hypotension and had required inotropic support (noradrenaline) to maintain adequate mean arterial pressure; as these episodes pre-dated her clozapine titration and there was no evidence of acute drop in blood pressure contemporaneous with clozapine administration, it was felt that continued titration was appropriate. She was extubated on day 32.

On day 44 she was noted to be ‘twitching’ by the medical team and a subsequent electroencephalogram confirmed non-convulsive status epilepticus. Her clozapine dose was reduced to 25 mg BD due to its effects on lowering the seizure threshold, and aripiprazole was also held for the same reason. She was commenced on levetiracetam with good effect. She was stepped down from ITU to critical care unit on day 49. Her trough steady state clozapine plasma concentration on day 50 (at 25 mg BD) was 80 ng/mL.

On day 67 she spiked a temperature of 38.0°C and became delirious; she was treated for urinary tract infection with co-amoxiclav; no further management was required for her delirium although her clozapine dose was held for one day. Clozapine dose was gradually increased to a total daily dose of 400 mg, with trough steady state plasma concentrations of 610 ng/mL on day 64. At the time of writing she was making a slow recovery in hospital.

## Discussion

The current pandemic has led to uncertainty regarding concurrent clozapine therapy in those with COVID-19; particularly in striking the balance between preventing relapse of schizophrenia with reducing risk of side effects and worsening of physical illness. In this retrospective case series, we have presented eight patients who were prescribed clozapine whilst sufficiently unwell with COVID-19 to require treatment in an acute medical hospital, including three who had stays in intensive care due to COVID-19 pneumonia. Our experience indicates that clozapine can be used safely and effectively in this patient group.

Clozapine remains the only antipsychotic with repeated proven efficacy in treatment-resistant schizophrenia.^12^ Switching to an alternative antipsychotic in patients established on clozapine is highly likely to lead to relapse, with potential consequences for the ability to continue treatment of the physical health condition.

Despite this, patients who take clozapine may be at particular risk from respiratory infections. Clozapine is the only antipsychotic that increases the likelihood of pneumonia in a dose-dependent relationship,^13^ with studies reporting a four-fold increase in risk.^14^ Pneumonia is a significant contributor to mortality in patients taking clozapine,^15^ and early data from one group have suggested an effect of clozapine on levels of some immunoglobulins leading to a reversible immunodeficiency-like syndrome, which may correlate with the rate of infections.^16^

Proposed mechanisms of this increased risk include clozapine-induced sedation and hypersalivation, which increases the risk of aspiration pneumonia. Furthermore, multiple medical conditions contribute to the risk of developing respiratory infections, many of which are common comorbidities in patients with treatment-resistant schizophrenia (particularly cardiovascular disease, diabetes and COPD).^17^ These medical conditions affected all the patients described in this case series to varying extents.

Clozapine is well-known to cause neutropenia and/or agranulocytosis in very rare cases,^18^ particularly towards the beginning of treatment.^19^ Although robust data have yet to emerge, there are early indications that severe COVID-19 infection features a lymphopenia,^20^ with both the severity of lymphopenia^21^ and the neutrophil to lymphocyte ratio^22^ possibly related to poorer outcomes in COVID-19 pneumonia. Data from our study indicated no neutropenia in our included patients. Taken together, we tentatively suggest that the potential clozapine-induced granulocyte suppression is unlikely to lead to worse outcomes in COVID-19 pneumonia, particularly in patients who have been maintained on clozapine for longer periods.

COVID-19 has multisystemic effects,^1^ and it has been suggested that some patients may be subject to a hyperinflammatory state mediated by massive cytokine release.^23^ This phenomenon, known as a cytokine storm, may lead us to expect high rates of delirium; and indeed, data so far have indicated that 9–20% of patients with COVID-19 experience delirium in the acute phase.^24,25^ In our study, the majority (five out of eight) of our patients experienced a delirium, which in many cases was prolonged.

Antipsychotics other than clozapine are often used for the management of the symptoms of delirium, although they do not treat the underlying delirium itself.^26^ Antipsychotics could prove useful in managing delirium in patients with COVID-19 pneumonia and ‘silent’ hypoxia,^27^ as they do not suppress the respiratory drive. In these cases, antipsychotics may be used for specific symptom-targeted treatment, for a defined short period of time, and at a minimally effective dose.^26^ As well as this, caution must be exercised when considering the addition of antipsychotics in patients already established on antipsychotic treatment, including clozapine.

There are several factors which may complicate clozapine dosing in patients with severe COVID-19 infection. Systemic inflammation can cause a reduction in metabolism of clozapine *via* hepatic CYP1A2 enzymes,^28^ which can cause a rise in clozapine plasma concentration; this may increase the severity of plasma concentration-related side effects such as sedation and hypersalivation, in turn increasing the risk of aspiration pneumonia. In addition, smoking is common in patients with severe mental illness,^29^ with cessation or reduction likely during respiratory infection. This reduction in the inhalation of the associated polycyclic aromatic hydrocarbons contributes to further increases in clozapine plasma concentrations *via* cessation of induction of CYP1A2, although this effect may not be immediately apparent.^26^

None of the patients in our case series had clozapine plasma concentrations measured on admission, but all except one had clozapine doses suspended temporarily or reduced at that point. We therefore suggest that, where possible, rapid analysis of clozapine concentrations in patients presenting with COVID-19 is essential to make informed decisions on the necessity and magnitude of dose reductions during the period of the infection, to avoid toxicity and exacerbation of adverse effects that may worsen the medical outcome. However, as clozapine serum levels may not be immediately available, we recommend dose reduction to be considered in those with a suspected inflammatory conditions such as COVID-19, due to potential increased risk of toxicity.^30^ We would also recommend that outside of medical settings, clinicians ensure that patients have up-to-date trough and steady-state serum clozapine levels as baseline, which will help to guide decisions on clozapine dosing in the event of COVID-19 infection.

We have presented three patients who required intubation in an intensive care unit due to COVID-19 pneumonia. There are currently no guidelines to suggest whether clozapine should be stopped or continued during sedation under these circumstances. In this case series, we described one intubated patient in which clozapine was continued at a reduced dose, and two in which clozapine was stopped for protracted periods with retitration initiated prior to ventilator weaning. Psychotic relapse occurred in those two who had their clozapine stopped. Further research is required into the consequences of both courses of action, but we tentatively suggest that safe continuation of clozapine in patients who are mechanically ventilated and/or sedated in intensive care settings is possible.

Involvement of liaison psychiatry expertise is essential to facilitate safe clozapine use in medical settings, ensuring appropriate dosing, side effect management and uninterrupted administration (including in patients who are intubated). Psychiatric and medical clinicians should be mindful of the risk of diagnostic overshadowing of the COVID-19 infection and remain alert for the rare, but potentially serious, adverse effects of clozapine in patients presenting to acute health services during the pandemic, such as clozapine-induced myocarditis.

Finally, ethnicity may play a role in poorer outcomes in COVID-19 infection, a finding which is supported by our small dataset. It is established that Black, Asian and Minority Ethnic (BAME) individuals are at increased risk of psychotic illnesses,^31^ and data from the COVID-19 pandemic are also suggesting that BAME people have poorer outcomes from SARS-CoV-2 infection.^32^ In our case series, three out of eight patients were Black, and of these two died; on the other hand, one out of five White patients died.

### Strengths

To our knowledge, this is the first study of its kind to assess outcomes in patients who had COVID-19 infection whilst inpatients in an acute medical hospital.

### Limitations

Our study has several limitations. The usual biases to a retrospective study design apply, including a preclusion of causation. Our patients had overall short length of follow-up, with the longest only 104 days; this was in part due to our view that the data required early publication. A well as this, patients known to our mental health trust on clozapine with COVID-19 who did not require hospital admission were not included in the case series. We did not include patient perspectives in this case series, in part due to practical limitation; we would support further research which includes patient and public engagement.

## Conclusion

The cases described here demonstrate that it is possible to continue clozapine therapy even in patients who are seriously affected by infection with COVID-19, and that doing so prevents a relapse in psychotic symptoms. Once clozapine has been excluded as a cause for the presenting symptoms, we believe it is usually reasonable to continue treatment, preferably using plasma concentrations to guide dosing. In our patients, serum clozapine concentrations were regretfully not always available. Where plasma concentrations are not available, we advise clinicians to assess the need for dose reduction considering factors including changes in smoking, likelihood and consequence of relapse, and the presence of signs of clozapine toxicity or plasma concentration-related adverse effects.

At the beginning of the pandemic, the uncertainty which surrounded clozapine use in severe COVID-19 use contributed to shared decisions to stop clozapine in patients included in this study, some of whom relapsed as a result. We hope our data help clinicians make more informed decisions on clozapine prescriptions in patients who become unwell with COVID-19, and that clinicians strongly consider continuing clozapine in patients who develop COVID-19.
